# Competing with big business: a randomised experiment testing the effects of messages to promote alcohol and sugary drink control policy

**DOI:** 10.1186/s12889-017-4972-6

**Published:** 2017-12-28

**Authors:** Maree Scully, Emily Brennan, Sarah Durkin, Helen Dixon, Melanie Wakefield, Colleen L. Barry, Jeff Niederdeppe

**Affiliations:** 1 Centre for Behavioural Research in Cancer, Cancer Council Victoria, 615 St Kilda Road, Melbourne, VIC 3004 Australia; 2 Department of Health Policy and Management, Johns Hopkins Bloomberg School of Public Health, 624 N. Broadway, Baltimore, MD 21205 USA; 3 Department of Communication, Cornell University, 476 Mann Library Building, Ithaca, NY 14853 USA

**Keywords:** Competitive framing, Health communication, Advocacy messages, Health policy, Health behaviour, Alcohol, Sugary drinks

## Abstract

**Background:**

Evidence-based policies encouraging healthy behaviours are often strongly opposed by well-funded industry groups. As public support is crucial for policy change, public health advocates need to be equipped with strategies to offset the impact of anti-policy messages. In this study, we aimed to investigate the effectiveness of theory-based public health advocacy messages in generating public support for sugary drink/alcohol policies (increased taxes; sport sponsorship bans) and improving resistance to subsequent anti-policy messages typical of the sugary drink/alcohol industry.

**Methods:**

We conducted a two-wave randomised online experiment assigning Australian adults to one of four health policies (sugary drink tax; sugary drink industry sports sponsorship ban; alcohol tax; alcohol industry sports sponsorship ban). Within each health policy, we randomised participants to one of five message conditions: (i) non-advocacy based message about the size and seriousness of the relevant health issue (control); (ii) standard pro-policy arguments alone; (iii) standard pro-policy arguments combined with an inoculation message (forewarning and directly refuting anti-policy arguments from the opposition); (iv) standard pro-policy arguments combined with a narrative message (a short, personal story about an individual’s experience of the health issue); or (v) standard pro-policy arguments combined with a composite inoculation and narrative message. At time 1, we exposed participants (*n* = 6000) to their randomly assigned message. Around two weeks later, we re-contacted participants (*n* = 3285) and exposed them to an anti-policy message described as being from a representative of the sugary drink/alcohol industry. Generalised linear models tested for differences between conditions in policy support and anti-industry beliefs at both time points.

**Results:**

Only the standard argument plus narrative message increased policy support relative to control at time 1. The standard argument plus narrative and standard argument plus inoculation messages were effective at increasing resistance to the persuasive impact of anti-policy messages relative to control at time 2.

**Conclusions:**

Dissemination of advocacy messages using inoculation or narrative components can help strengthen public resistance to subsequent anti-policy messages from industry groups.

**Electronic supplementary material:**

The online version of this article (10.1186/s12889-017-4972-6) contains supplementary material, which is available to authorized users.

## Background

Framing of policy issues in popular discourse is often competitive, with public health advocates and industry groups offering contrasting views on the causes of a problem and/or the actions required to solve it [1, 2]. The tobacco, food, beverage, and alcohol industries direct substantial resources into lobbying against policy reforms that would have an adverse impact on their commercial interests, making it difficult for public health advocates to compete in terms of the volume of exposure [3–5]. As public support is crucial for policy change [6], advocates need to be equipped with strategies to offset the strong opposing arguments propagated by industry groups.

Two theory-based approaches may offer utility in these efforts. First, inoculation messages operate on the premise that people can be protected from future attempts at persuasion by messages that warn them of the impending threat to their attitude and refute anticipated opposing arguments [7, 8]. Key mechanisms posited to be involved in the inoculation process include an increase in perceptions of threat (in this study conceptualised as a threat to the freedom to make up one’s own mind about an issue) and greater counter-arguing against persuasive attacks when they are subsequently encountered [9]. A meta-analysis found inoculation messages to be more effective than one-sided messages (i.e. those that only present arguments in support of a proposition) or no-exposure controls at increasing resistance to subsequent counter-frames [10]. Inoculation effects can occur irrespective of the direction and strength of pre-existing attitudes toward an issue [11]. While originally designed to confer resistance to counter-messages, inoculation messages may also provide an initial persuasive effect by positively influencing attitudes and beliefs immediately following exposure [8].

Second, narrative messages take the form of a story and focus on the experiences of one or more characters [12]. Narrative messages may lead to persuasion by generating emotional connections with story characters and/or creating high levels of story engagement [13]. The process of being deeply engaged with a narrative can affect audiences by increasing emotional responding and reducing negative cognitive responses (such as counter-arguing) to the message [14]. The persuasive impact of narratives can increase over time, suggesting they may be well-suited for competitive framing situations [15, 16]. Two recent meta-analyses found that narratives have persuasive effects on changes in attitudes, intentions and behaviour [17, 18].

Prior studies conducted in the United States have examined the impact and resilience of separate, print-based inoculation [19, 20] and narrative [20] messages on policy support within a competitive message environment. The present study builds on this research by testing the impact of public health advocacy messages that include inoculation and narrative persuasive components, both separately and in combination, in Australia. To assess the potential benefits for advocates of using inoculation and/or narrative strategies when promoting health policy changes, we compared these messages to a standard advocacy message and to a control message (non-advocacy based message about the relevant issue). We delivered all messages as a simulated radio segment in light of evidence suggesting that audio and video messages, which are common modes of communication for public health advocates, can produce stronger effects for narratives than messages delivered via text [17].

Two key areas of current policy debate between public health and industry centre on preventing obesity and reducing alcohol-related harms, providing an opportunity to study the efficacy of advocacy messages across multiple topics. Across both issues, public health advocates have emphasised the need for population-level approaches requiring government intervention, such as taxation and restrictions on advertising and sponsorship for unhealthy foods and beverages [21]. In response, these industries typically highlight the role of personal responsibility and attempt to position themselves as being ‘part of the solution’ by promoting self-regulation [22, 23]. The contribution of sugary drinks in driving obesity rates [24] has been a particular point of contention, with the sugary drink industry employing ‘social responsibility’ campaigns to protect against claims from the health sector that their products pose serious health concerns and should be subject to stronger legislative controls [25, 26].

The present study aims to assess the effectiveness of advocacy messages containing inoculation and/or narrative components in (a) generating support for four different health policies related to sugary drinks/alcohol, and (b) improving resistance to subsequent anti-policy messages typical of the sugary drink/alcohol industries. Based on theory and prior research, we hypothesise that inoculation and narrative messages (delivered separately and in combination) will produce immediate persuasive effects by increasing policy support and anti-industry beliefs relative to the control message directly after exposure (H1). We further predict that exposure to an inoculation message will provide protective effects by reducing the impact of a subsequent industry anti-policy message, resulting in greater policy support and stronger anti-industry beliefs compared to exposure to the control message at a two-week follow-up (H2). Unlike inoculation messages, narratives are not specifically designed to confer resistance to counter-messages. However, given evidence that the persuasive effects of narrative messages can increase over time [15, 16], we test whether exposure to an advocacy message that only incorporates narrative components offsets the impact of a subsequent industry anti-policy message, delivered around two weeks later (RQ1). We further explore whether inoculation and/or narrative effects are specific to the health policy being explicitly addressed in the message or if they spill-over to other policies related to the same health issue (RQ2). Finally, we test whether inoculation and/or narrative messages produce stronger effects than a standard advocacy message (RQ3).

## Method

### Study design

We conducted a two-wave between-subjects online experiment whereby we randomly assigned Australian adults to one of four public health policies: (a) a 20% tax on sugary drinks; (b) removal of sugary drink sponsorship from sport; (c) a volume-based tax on alcohol; or (d) removal of alcohol sponsorship from sport. Within each health policy, we randomised participants to one of five message conditions: (i) non-advocacy based message about the size and seriousness of the relevant issue (control); (ii) standard pro-policy arguments (Standard); (iii) Standard + inoculation (Standard + I); (iv) Standard + narrative (Standard + N); (v) Standard + inoculation + narrative (Standard + I + N). At time 1 (t1), we exposed participants to their randomly assigned message and then asked them questions assessing policy support and anti-industry beliefs to test for immediate persuasive effects of the advocacy messages. Around two weeks later (time 2 (t2)), we exposed participants to an anti-policy message opposing the proposed health policy, then re-assessed policy support and anti-industry beliefs to test for protective effects of the advocacy messages.

We presented all messages in audio format as mock radio segments. We wrote and recorded all messages specifically for the study, with content informed by policy statements and other public materials (including news articles) on both sides of the four health policy debates.

### Message conditions

All participants heard a short statement read by a radio presenter introducing obesity (sugary drink policies) or alcohol-related harms (alcohol policies) as a major issue in Australia before listening to their assigned message at t1. Participants in the control condition heard nothing other than this introductory statement (mean length across the four health policies: 18.5 s; range: 15–22 s). In the Standard condition, the introductory statement was followed by an interview with a fictional public health advocate who presented arguments for why the particular policy proposal should be introduced in Australia (mean length: 186.5 s; range: 175–202 s). In the Standard + I condition, additional interviewer questions allowed the public health advocate to deliver the inoculation components. The inoculation components included (a) warning the listener that the sugary drink/alcohol industry will try to persuade them to oppose the policy, (b) illuminating the practices and motives of the industry and emphasising that they cannot be trusted, and (c) refuting anticipated industry arguments against the policy (mean length: 246.3 s; range: 230–256 s). The Standard + N messages featured interviews with both the public health advocate presenting the standard pro-policy arguments and a fictional mother relating her own personal story (i.e. narrative). The mother described her efforts to protect her teenage daughter from harms associated with sugary drink/alcohol consumption, and how they were being undermined by the marketing of these products and their low cost and ready accessibility (mean length: 308.5 s; range: 290–330 s). The Standard + I + N messages included the public health advocate presenting the standard advocacy and inoculation components, and the mother telling her personal story (mean length: 368.3 s; range: 344–386 s) (See Additional file 1 for written transcripts of the four Standard + I + N advocacy messages).

### Anti-policy messages

The industry-based anti-policy messages at t2 featured an interview with a fictional industry spokesperson who highlighted the complexity of the obesity/alcohol issue and presented arguments against the proposed policy, including those related to the concepts of government intrusion, self-regulation and personal responsibility (mean length: 145 s; range: 131–159 s) (See Additional file 2 for written transcripts of the four industry anti-policy messages).

### Participants

A sampling frame of Australian adults was sourced primarily through a commercial data collection agency’s (I-view’s) national online panel, with supplementary sample obtained from a partner panel (Research Now). Both are non-probability based online panels comprising members initially sourced in a variety of ways such as computer-assisted telephone interviews, face-to-face research and online market research databases, and offer members points for completing surveys that can be redeemed for a variety of rewards such as gift cards. Applying broad age, sex and location quotas, random samples of panel members were sent an email invitation to participate along with a link to the survey. Informed consent was provided by all participants, after having read the study information, by clicking to proceed with completing the survey. We obtained ethical approval for the study (including the consent procedure) from Cancer Council Victoria’s Institutional Research Review Committee (IER 1505). Based on results obtained from a previous experimental study examining inoculation effects on public support for taxes on sugary drinks in the United States [19], we estimated that a sample size of 3200 adults at t2 would be sufficient to detect effects (at 80% power).

Email invitations were sent to 190,438 panellists between 9th September and 16th November 2015, and of these recipients, 11,316 clicked to commence the screening questions (response rate: 5.9%). Panellists were ineligible to participate in the study if they were aged under 18 years (*n* = 45), worked in related industries such as marketing, advertising, health promotion, market research, sugary drinks or alcohol manufacturing, distribution and supply (*n* = 241), were unable to hear the audio check question (*n* = 309), were identified as duplicates (*n* = 149), or their demographic quota had already been reached (*n* = 2391). Of the 8181 eligible panellists who commenced the t1 survey, 1614 were excluded due to abandoning the survey before completion while 567 were removed following standard quality control processes, resulting in a final t1 sample of 6000 participants.

Approximately 2 weeks later (average time between surveys = 15.8 days; range: 2–42 days), I-view re-contacted t1 study participants by email and asked them to complete a follow-up survey. A total of 3285 panellists completed the t2 survey between 16th September and 25th November (follow-up rate: 54.8%). Reasons for non-completion included: not clicking on or receiving the survey link within the t2 survey period (*n* = 1265); being screened out at the audio check question (*n* = 359); abandoning the survey before completion (*n* = 999); and technical or quality assurance issues (*n* = 92). Table 1 shows the t1 and t2 samples were comparable by randomised condition and on all demographic characteristics except age group, with the t2 sample comprised of slightly older participants. Both samples were skewed towards females [27] and older adults [27] but were similar to the Australian population in terms of socio-economic status [28].

**Table 1:** Sample characteristics at Time 1 (*n* = 6000) and Time 2 (*n* = 3285)

	Time 1 (t1) % (*n*)	Time 2 (t2) % (*n*)	Test statistic
*Health policy assignment*			
20% tax on sugary drinks	25.0 (1500)	24.9 (819)	Χ^2^(3) = 0.09, *p* = .992
Removal of sugary drink sponsorship from sport	25.0 (1500)	25.0 (821)	
Volume-based tax on alcohol	25.0 (1500)	24.8 (815)	
Removal of alcohol sponsorship from sport	25.0 (1500)	25.3 (830)	
*Message condition*			
Control	20.0 (1200)	19.6 (644)	Χ^2^(4) = 0.32, *p* = .988
Standard pro-policy arguments (Standard)	20.0 (1200)	20.3 (667)	
Standard + inoculation	20.0 (1200)	19.9 (655)	
Standard + narrative	20.0 (1200)	20.2 (664)	
Standard + inoculation + narrative	20.0 (1200)	19.9 (655)	
*Sex*			
Male	40.8 (2446)	42.2 (1386)	Χ^2^(1) = 1.78, *p* = .182
Female	59.2 (3554)	57.8 (1899)	
*Age (years)*			
18–24	7.5 (450)	5.9 (193)	Χ^2^(5) = 13.60, *p* = .018
25–34	14.2 (853)	13.4 (441)	
35–44	14.1 (847)	13.6 (448)	
45–54	15.5 (929)	15.3 (504)	
55–64	21.5 (1292)	22.5 (739)	
65 and older	27.2 (1629)	29.2 (960)	
*SES (area-based)* ^*a*^			
Quintile 1 (high disadvantage)	16.2 (974)	16.4 (539)	Χ^2^(4) = 1.34, *p* = .855
Quintile 2	18.2 (1090)	18.3 (602)	
Quintile 3	19.2 (1151)	18.2 (598)	
Quintile 4	22.4 (1342)	22.6 (743)	
Quintile 5 (low disadvantage)	24.0 (1442)	24.4 (802)	
*Highest level of education*			
Some secondary school or less	12.4 (744)	13.2 (433)	Χ^2^(4) = 1.34, *p* = .854
Finished secondary school	17.6 (1053)	17.7 (582)	
Some tertiary education	26.0 (1561)	25.6 (840)	
Finished tertiary education	29.5 (1768)	29.1 (956)	
Higher degree/diploma	14.6 (874)	14.4 (474)	
*Parent/carer of child aged under 18*			
No	76.4 (4584)	77.7 (2552)	Χ^2^(1) = 1.98, *p* = .160
Yes	23.6 (1416)	22.3 (733)	
*Body mass index (BMI) category* ^*b*^			
Underweight	2.7 (139)	2.5 (70)	Χ^2^(3) = 0.84, *p* = .839
Healthy weight	37.3 (1926)	38.1 (1079)	
Overweight	32.6 (1682)	32.1 (907)	
Obese	27.5 (1420)	27.3 (773)	
*Consumed sugary drinks in past 7 days* ^*c*^			
No	48.4 (1453)	50.6 (830)	Χ^2^(1) = 2.01, *p* = .156
Yes	51.6 (1547)	49.4 (810)	
*Frequency of drinking alcohol in last 12 months* ^*d*^			
Never / non-drinker	16.1 (484)	16.2 (267)	Χ^2^(2) = 0.16, *p* = .922
2–3 days a month or less	40.8 (1225)	41.3 (680)	
At least 1–2 days a week	43.0 (1291)	42.4 (698)	
*Political ideology (1 = Left to 10 = Right)*			
Mean (sd)	5.43 (1.93)	5.44 (1.93)	F(1) = 0.09, *p* = .767

Note: Percentages are rounded so may not sum to 100%

^a^SES was determined according to the Australian Bureau of Statistic’s Index of Relative Socio-Economic Disadvantage ranking for Australia using participant’s residential postcode [34]. Data is missing for 1 participant at t1 and t2 who provided an invalid postcode

^b^BMI was computed using participant’s self-reported height and weight [BMI = weight (kg) / height (m)^2^] and collapsed into categories according to World Health Organization definitions [35]. BMI information is missing for 833 participants at t1 and 456 participants at t2 as they did not self-report their height and/or weight

^c^This question was only asked of participants assigned to a sugary drink policy (t1: *n* = 3000; t2: *n* = 1640)

^d^This question was only asked of participants assigned to an alcohol policy (t1: n = 3000; t2: *n* = 1645)

### Outcome measures

#### Policy support

We asked participants to indicate their level of support (1 = *strongly oppose* to 7 = *strongly support*) for their assigned policy (‘target policy’) and two other policies (‘non-target policies’) within the same health domain. The three sugary drink policies assessed were: “A 20 percent tax on sugary drinks”; “Removing sugary drink sponsorship from sport”; and “Requiring large and prominently placed health warning labels on sugary drinks”. The three alcohol policies were: “A volume-based tax on alcohol products so that all drinks are taxed according to their alcohol content”; “Removing alcohol sponsorship from sport”; and “Requiring large and prominently placed health warning labels on alcohol containers”.

#### Anti-industry beliefs

Using items adapted from Niederdeppe et al. [19, 20], we asked participants assigned to a sugary drink policy to indicate the extent to which they agreed or disagreed (1 = *strongly disagree* to 7 = *strongly agree*) that sugary drink companies: “deny that sugary drinks cause obesity”; “only care about making a lot of money”; “try to get young people to drink sugary drinks”. We asked those assigned to an alcohol policy about their beliefs that alcohol companies: “deny they market their products to young people”; “only care about making a lot of money”; “try to get young people to drink alcohol”. The three items averaged to form acceptable scales for each health domain [sugary drinks: Cronbach’s α = .78 (t1) and .80 (t2); alcohol: α = .70 (t1) and .68 (t2)].

To control for possible order effects, we rotated the order in which participants completed the policy support and anti-industry beliefs question modules and randomised the presentation of items within each module.

### Manipulation checks

Prior to assessment of the two outcome measures at t1, participants assigned to one of the four advocacy messages completed additional questions related to proposed mechanisms of inoculation (i.e. perceived threat to freedom) and narratives (i.e. counter-arguing of message) as manipulation checks.

#### Perceived threat to freedom

We asked participants about the degree to which they anticipated that [soft drink/alcohol] companies pose a threat to one’s freedom to decide, “how we as a society should deal with the problem of [obesity/alcohol-related harm].” Using items originally adapted from Dillard and Shen [29] and subsequently used by Niederdeppe et al. [20], participants reported the extent to which they agreed or disagreed (1 = *strongly disagree* to 7 = *strongly agree*) that [soft drink/alcohol] companies will: “try to tell me what to think”; “try to make up my mind for me”; “try to manipulate my thoughts”; “try to pressure me”. We then averaged these four items together to form reliable scales for each health domain (sugary drinks: Cronbach’s α = .95; alcohol: α = .96).

#### Counter-arguing

We asked participants four questions (two worded positively and two worded negatively), to assess the degree to which they engaged in counter-arguing after listening to the interviewee/s’ views on the [obesity/alcohol] issue. Items were from Niederdeppe et al. [20] (originally adapted from Miller et al. [30]) and used the same 7-point Likert scales (1 = *strongly disagree* to 7 = *strongly agree*). The two positive items (“I found myself agreeing with the interviewee/s’ points” and “I accepted a lot of the arguments the interviewee/s offered”) were reverse-coded and combined with the two negative items (“I found myself disagreeing with the interviewee/s’ points” and “I thought of a lot of arguments against what the interviewee/s were saying”) to form acceptable scales for each health domain (Cronbach’s α = .86 for both sugary drinks and alcohol).

The exact wording of our survey items and their corresponding response scales are provided in Additional file 3.

### Statistical analysis

We analysed data using Stata/MP V.14.2 [31]. We performed chi-square (for categorical variables) and analysis of variance (for continuous variables) tests to assess whether random assignment to message condition yielded equivalent demographic groups. Since the distribution of age group was significantly different across message conditions at t1 (χ^2^(20) = 37.02, *p* = .012), we included age group as a covariate in all models.

We used linear regression to test for differences between message conditions in (a) target policy support, (b) anti-industry beliefs, and (c) average non-target policy support at t1 and t2 respectively. Models presented in the tables use the control message condition as the reference category; where the standard advocacy message is the comparison (RQ3), we re-ran the models with the Standard condition as the reference category (findings reported in text only). We included number of days elapsed between surveys as a covariate in the t2 analyses as evidence suggests inoculation effects begin to decay after two weeks [10]. We initially ran all models including interaction terms between message condition and health policy assignment and conducted a global test of the significance of this interaction after each model. As none were significant, we omitted these interaction terms from the final models and interpreted message effects as equivalent across policies. However, we included health policy assignment as a covariate in all models. Means and standard deviations for each outcome measure at t1 and t2 by message condition and health policy assignment are provided in a supplementary table (see Additional file 4).

For exploratory purposes, we examined interactions between message condition and three individual characteristics of particular interest—parental status (parent/carer of child aged under 18 or not), body mass index (underweight/healthy weight vs. overweight/obese), and sugary drink/alcohol consumption (weekly consumer or not)—for each of our final models. None of these interactions were statistically significant, so for brevity these results are not reported.

## Results

### Manipulation checks

Linear regression analyses conducted on the measures assessed in the advocacy message conditions as manipulation checks (controlling for health policy assignment and age group) indicate that inclusion of inoculation components in the advocacy message did successfully induce higher levels of perceived threat to freedom among participants (Standard + I: M = 4.67, sd = 1.60; B = 0.14, β = 0.04, *p* = .025; Standard + I + N: M = 4.72, sd = 1.55; B = 0.19, β = 0.05, *p* = .003) compared to a standard advocacy message (M = 4.54, sd = 1.54). However, we note that participants who received a narrative message without the inoculation components also reported higher levels of perceived threat to freedom than those in the Standard condition (Standard + N: M = 4.67, sd = 1.50; B = 0.14, β = 0.04, *p* = .021).

Contrary to expectations based on theory, the presence of a narrative in the advocacy message did not reduce counter-arguing of the message. Indeed there was evidence that participants in the Standard + I + N condition actually engaged in more counter-arguing than those in the Standard condition (Standard: M = 3.00, sd = 1.32; Standard + I + N: M = 3.16, sd = 1.30; B = 0.15, β = 0.05, *p* = .006), while participants in the Standard + N condition engaged in similar levels of counter-arguing as those in the Standard condition (Standard + N: M = 3.08, sd = 1.23; B = 0.06, β = 0.02, *p* = .252). Although participants’ level of counter-arguing was higher in the Standard + I condition compared to the Standard condition, this difference was non-significant (Standard + I: M = 3.12, sd = 1.41; B = 0.10, β = 0.03, *p* = .057).

### Effects of advocacy messages on policy support and anti-industry beliefs at time 1

Table 2 shows that participants exposed to a Standard + N advocacy message had greater target policy support than those who received a control message at t1 (B = 0.19, β = 0.04, *p* = .012). Exposure to the Standard + I or Standard + I + N advocacy messages did not significantly increase target policy support relative to the control message. There were no significant differences in anti-industry beliefs between the control group and each of the inoculation and/or narrative message conditions. Combined, these results provide limited support for our hypothesis (H1) that inoculation and/or narrative messages would produce immediate persuasive effects.

**Table 2:** Linear regression models testing message effects on policy support and anti-industry beliefs at Time 1 (*n* = 6000)

	Time 1 outcome measures								
	Target policy support^a^			Anti-industry beliefs^b^			Average non-target policy support^c^		
	B (95% CI)	β	*p*	B (95% CI)	β	*p*	B (95% CI)	β	*p*
Message condition									
Control	Ref			Ref			Ref		
Standard pro-policy arguments (Standard)	0.11 (−0.03, 0.25)	0.02	.137	−0.05 (−0.14, 0.04)	−0.02	.263	−0.02 (−0.14, 0.09)	−0.01	.691
Standard + inoculation	0.09 (−0.06, 0.23)	0.02	.249	0.04 (−0.05, 0.13)	0.01	.363	0.02 (−0.09, 0.13)	0.01	.748
Standard + narrative	**0.19 (0.04, 0.33)**	**0.04**	**.012**	−0.03 (−0.11, 0.06)	−0.01	.573	0.06 (−0.05, 0.18)	0.02	.275
Standard + inoculation + narrative	0.10 (−0.04, 0.25)	0.02	.157	0.03 (−0.06, 0.12)	0.01	.508	−0.07 (−0.18, 0.05)	−0.02	.248
Covariates									
Health policy assignment									
20% tax on sugary drinks	Ref			Ref			Ref		
Removal of sugary drink sponsorship from sport	**0.73 (0.60, 0.86)**	**0.17**	**<.001**	**0.10 (0.02, 0.17)**	**0.04**	**.018**	**−0.39 (−0.49, −0.29)**	**−0.12**	**<.001**
Volume-based tax on alcohol	0.11 (−0.02, 0.24)	0.03	.094	**−0.18 (−0.26, −0.11)**	**−0.07**	**<.001**	−0.07 (−0.17, 0.04)	−0.02	.205
Removal of alcohol sponsorship from sport	**0.72 (0.59, 0.85)**	**0.17**	**<.001**	**−0.15 (−0.23, −0.07)**	**−0.06**	**<.001**	**−0.44 (−0.54, −0.34)**	**−0.13**	**<.001**
Age (years)									
18–24	Ref			Ref			Ref		
25–34	**0.29 (0.08, 0.49)**	**0.05**	**.007**	**0.15 (0.03, 0.28)**	**0.05**	**.017**	0.12 (−0.04, 0.28)	0.03	.136
35–44	**0.32 (0.11, 0.53)**	**0.06**	**.002**	**0.24 (0.12, 0.37)**	**0.08**	**<.001**	0.16 (−0.00, 0.32)	0.04	.057
45–54	0.11 (−0.10, 0.31)	0.02	.301	**0.39 (0.26, 0.51)**	**0.13**	**<.001**	**0.16 (0.01, 0.32)**	**0.04**	**.042**
55–64	**0.50 (0.31, 0.70)**	**0.11**	**<.001**	**0.47 (0.35, 0.59)**	**0.17**	**<.001**	**0.34 (0.19, 0.49)**	**0.10**	**<.001**
65 and older	**0.82 (0.64, 1.01)**	**0.20**	**<.001**	**0.54 (0.43, 0.66)**	**0.21**	**<.001**	**0.60 (0.45, 0.75)**	**0.19**	**<.001**

*B* unstandardised regression coefficient; *CI* confidence interval; *β* standardised regression coefficient; *Ref* referent category in linear regression model. Boldfaced results are significant at *p* < .05

^a^Participants’ level of support for their assigned policy at t1 which was recorded on a 7-point scale (1 = *strongly oppose* to 7 = *strongly support*)

^b^For participants assigned to a sugary drink policy, the anti-industry beliefs measured were that sugary drink companies: “deny that sugary drinks cause obesity”; “only care about making a lot of money”; “try to get young people to drink sugary drinks”. For participants assigned to an alcohol policy, the anti-industry beliefs measured were that alcohol companies: “deny they market their products to young people”; “only care about making a lot of money”; “try to get young people to drink alcohol”. Participants’ level of agreement with their three anti-industry beliefs at t1 were recorded on 7-point scales (1 = *strongly disagree* to 7 = *strongly agree*), and subsequently averaged to create this outcome measure

^c^For participants assigned to the “20% tax on sugary drinks” policy, the two non-targeted policies were removal of sugary drink sponsorship from sport and health warning labels on sugary drinks. For participants assigned to the “removal of sugary drink sponsorship from sport” policy, the two non-targeted policies were a 20% tax on sugary drinks and health warning labels on sugary drinks. For participants assigned to the “volume based tax on alcohol” policy, the two non-targeted policies were removal of alcohol sponsorship from sport and health warning labels on alcohol containers. For participants assigned to the “removal of alcohol sponsorship from sport” policy, the two non-targeted policies were a volume based tax on alcohol and health warning labels on alcohol containers. Participants’ level of support for their two non-targeted policies at t1 were recorded on 7-point scales (1 = *strongly oppose* to 7 = *strongly support*), and subsequently averaged to create this outcome measure

There was no evidence of spill-over effects at t1 (RQ2), with no differences in levels of support for non-targeted policies between those who received the Standard + I, Standard + N or Standard + I + N messages relative to the control group. Additional exploratory analyses directly comparing the inoculation and/or narrative messages to the standard advocacy message (RQ3, results not shown in Table 2) indicated that participants exposed to a Standard + I, Standard + N or Standard + I + N message did not have greater policy support at t1 than those who received a Standard message alone (all *p* > .05). Participants exposed to a Standard + I message did, however, hold stronger anti-industry beliefs compared to the Standard message group (B = 0.09, β = 0.03, *p* = .042).

### Effects of advocacy messages on policy support and anti-industry beliefs at time 2

Among t2 respondents, target policy support (t1: M = 4.87, sd = 1.86; t2: M =3.79, sd = 1.94), anti-industry beliefs (t1: M = 5.47, sd = 1.11; t2: M = 5.06, sd = 1.21), and average non-target policy support (t1: M = 5.10, sd = 1.44; t2: M = 4.75, sd = 1.49) were all weaker following exposure to the industry anti-policy message about two weeks after their original message exposure (advocacy or control message). We observed these declines across all conditions; the difference between t1 and t2 means ranged from 0.97–1.25 for target policy support, 0.35–0.54 for anti-industry beliefs, and 0.27–0.53 for average non-target policy support.

However, Table 3 shows participants originally exposed to a message that incorporated inoculation components (either with or without a narrative) had greater target policy support at t2 than those who had previously only received a control message (Standard + I: B = 0.31, β = 0.06, *p* = .003; Standard + I + N: B = 0.28, β = 0.06, *p* = .009). In further support of our hypothesis (H2) that prior exposure to an inoculation message would provide protective effects against a subsequent industry anti-policy message, participants who had previously received a Standard + I message also held stronger anti-industry beliefs at t2 compared to the control group (B = 0.16, β = 0.05, *p* = .019). Contrary to expectations, though, there was no significant difference in anti-industry beliefs between the combined inoculation and narrative message condition (Standard + I + N) and the control message condition (B = 0.07, β = 0.02, *p* = .268). With regard to testing whether the message that only incorporated narrative components provided similar protective effects (RQ1), participants originally exposed to a Standard + N message also had greater policy support (B = 0.32, β = 0.07, *p* = .002) and held stronger anti-industry beliefs (B = 0.14, β = 0.05, *p* = .034) at t2 than those in the control group.

**Table 3:** Linear regression models testing message effects on policy support and anti-industry beliefs at Time 2 (*n* = 3285)

	Time 2 outcome measures								
	Target policy support^a^			Anti-industry beliefs^b^			Average non-target policy support^c^		
	B (95% CI)	β	*p*	B (95% CI)	β	*p*	B (95% CI)	β	*p*
Message condition									
Control	Ref			Ref			Ref		
Standard pro-policy arguments (Standard)	**0.26 (0.06, 0.47)**	**0.05**	**.013**	0.08 (−0.05, 0.21)	0.03	.255	**0.16 (0.01, 0.32)**	**0.04**	**.041**
Standard + inoculation	**0.31 (0.10, 0.52)**	**0.06**	**.003**	**0.16 (0.03, 0.29)**	**0.05**	**.019**	**0.22 (0.06, 0.38)**	**0.06**	**.007**
Standard + narrative	**0.32 (0.11, 0.53)**	**0.07**	**.002**	**0.14 (0.01, 0.27)**	**0.05**	**.034**	**0.27 (0.11, 0.43)**	**0.07**	**.001**
Standard + inoculation + narrative	**0.28 (0.07, 0.49)**	**0.06**	**.009**	0.07 (−0.06, 0.20)	0.02	.268	0.11 (−0.05, 0.27)	0.03	.181
Covariates									
Health policy assignment									
20% tax on sugary drinks	Ref			Ref			Ref		
Removal of sugary drink sponsorship from sport	**0.26 (0.08, 0.45)**	**0.06**	**.006**	**−0.15 (−0.26, −0.03)**	**−0.05**	**.013**	**−0.47 (−0.61, −0.33)**	**−0.14**	**<.001**
Volume-based tax on alcohol	**0.21 (0.03, 0.40)**	**0.05**	**.025**	**0.18 (0.06, 0.29)**	**0.06**	**.003**	**0.32 (0.18, 0.47)**	**0.09**	**<.001**
Removal of alcohol sponsorship from sport	**0.52 (0.34, 0.71)**	**0.12**	**<.001**	−0.10 (−0.22, 0.01)	−0.04	.086	−0.09 (−0.23, 0.05)	−0.03	.195
Age (years)									
18–24	Ref			Ref			Ref		
25–34	0.28 (−0.04, 0.60)	0.05	.091	0.16 (−0.04, 0.36)	0.05	.121	0.16 (−0.09, 0.40)	0.04	.210
35–44	0.15 (−0.18, 0.47)	0.03	.371	0.11 (−0.09, 0.31)	0.03	.287	0.04 (−0.21, 0.28)	0.01	.764
45–54	−0.28 (−0.60, 0.03)	−0.05	.080	0.12 (−0.08, 0.32)	0.04	.238	−0.05 (−0.29, 0.19)	−0.01	.701
55–64	−0.15 (−0.45, 0.16)	−0.03	.343	0.12 (−0.07, 0.31)	0.04	.211	0.14 (−0.09, 0.38)	0.04	.223
65 and older	−0.13 (−0.43, 0.16)	−0.03	.374	0.07 (−0.11, 0.26)	0.03	.433	**0.29 (0.06, 0.51)**	**0.09**	**.013**
Days elapsed between surveys	**−0.01 (−0.02, −0.01)**	**−0.06**	**<.001**	**−0.01 (−0.01, −0.00)**	**−0.06**	**.001**	−0.01 (−0.01, 0.00)	−0.03	.061

*B* unstandardised regression coefficient; *CI* confidence interval; *β* standardised regression coefficient; *Ref* referent category in linear regression model. Boldfaced results are significant at *p* < .05

^a^Participants’ level of support for their assigned policy at t2 which was recorded on a 7-point scale (1 = *strongly oppose* to 7 = *strongly support*)

^b^For participants assigned to a sugary drink policy, the anti-industry beliefs measured were that sugary drink companies: “deny that sugary drinks cause obesity”; “only care about making a lot of money”; “try to get young people to drink sugary drinks”. For participants assigned to an alcohol policy, the anti-industry beliefs measured were that alcohol companies: “deny they market their products to young people”; “only care about making a lot of money”; “try to get young people to drink alcohol”. Participants’ level of agreement with their three anti-industry beliefs at t2 were recorded on 7-point scales (1 = *strongly disagree* to 7 = *strongly agree*), and subsequently averaged to create this outcome measure

^c^For participants assigned to the “20% tax on sugary drinks” policy, the two non-targeted policies were removal of sugary drink sponsorship from sport and health warning labels on sugary drinks. For participants assigned to the “removal of sugary drink sponsorship from sport” policy, the two non-targeted policies were a 20% tax on sugary drinks and health warning labels on sugary drinks. For participants assigned to the “volume based tax on alcohol” policy, the two non-targeted policies were removal of alcohol sponsorship from sport and health warning labels on alcohol containers. For participants assigned to the “removal of alcohol sponsorship from sport” policy, the two non-targeted policies were a volume based tax on alcohol and health warning labels on alcohol containers. Participants’ level of support for their two non-targeted policies at t2 were recorded on 7-point scales (1 = *strongly oppose* to 7 = *strongly support*), and subsequently averaged to create this outcome measure

There was some evidence of spill-over effects at t2 (RQ2), with average non-target policy support significantly higher among participants with prior exposure to a Standard + I (B = 0.22, β = 0.06, *p* = .007) or Standard + N (B = 0.27, β = 0.07, *p* = .001) message. However, Standard + I + N messages did not produce greater support for non-targeted policies than the control group (B = 0.11, β = 0.03, *p* = .181). Direct comparisons between the inoculation and/or narrative messages and the standard advocacy message (RQ3) indicated that participants originally exposed to the Standard + I, Standard + N or Standard + I + N messages had comparable levels of policy support and anti-industry beliefs as participants who had previously received only the Standard message alone (all *p* > .05; results not shown in Table 3). We also note that the Standard condition produced higher target (B = 0.26, β = 0.05, *p* = .013) and non-target (B = 0.16, β = 0.04, *p* = .041) policy support at t2 compared to the control condition, but anti-industry beliefs in the Standard and control groups were not statistically different (B = 0.08, β = 0.03, *p =* .255; see Table 3).

## Discussion

Our results suggest that advocacy messages incorporating inoculation or narrative components can increase public resilience to subsequent anti-policy messages. Across two policy domains (sugary drinks and alcohol) and tactics (taxation and sport sponsorship bans), inoculation and narrative messages *delivered separately* were successful in countering the persuasive impact of opposing arguments from the sugary drink and alcohol industry as measured by participants’ level of support for the health policy being addressed in the message and the strength of their anti-industry beliefs after exposure to the anti-policy message (at t2, around two weeks later).

There was limited evidence of immediate effects of the inoculation and narrative messages above and beyond a message about the size and seriousness of the health issue (i.e. the control message), with only the narrative condition leading to higher target policy support after initial exposure. While this finding may appear disappointing at first glance, it does highlight the need to consider experimental designs that accommodate exposure to competing arguments and assessment of responses at multiple time points relative to initial, single message exposure when testing the potential impact of advocacy messages in the real world. As noted earlier, public debates about policies targeting powerful industries (like the sugary drink and alcohol industries) are almost certain to feature widespread exposure to anti-policy arguments propagated by these industries. It is therefore essential to identify message strategies that are successful at countering these anti-policy arguments over time.

Our observation that narrative messages provided resistance to a subsequent anti-policy message runs counter to one previous study [20] but is congruent with other experimental work showing delayed persuasive effects of fictional narratives, albeit in the absence of competition from opposing messages [15, 16]. It is possible that the strong performance of the narrative messages in the present study stems from the fact they induced increased levels of perceived threat to freedom among participants (as per the inoculation message). While the narrative messages were not intentionally designed to influence perceptions of threat to freedom, they may have prompted this response from participants due to the mother describing how her efforts to protect her daughter were being undermined by industry marketing. However, as all participants received the industry anti-policy message at t2, we are unable to separate out protective effects from persuasive effects that grow over time. The mode of delivery may also have been a contributing factor, with suggestions that audio narrative messages are better able to evoke emotions and transport audiences into the story compared to print-based messages, which may be more conducive to rational processing [17]. Research that involves manipulation of both the delivery channel and timing of subsequent exposure to the competing message is needed to better understand the comparative short- and long-term effects of print- and audio-based narrative messages in the face of opposing arguments.

Overall, policy support and anti-industry beliefs were lower following exposure to the industry anti-policy message at t2. The inability of the inoculation and/or narrative messages to fully offset the persuasive impact of opposing arguments from the sugary drink and alcohol industry is likely reflective of the strength and recency of the anti-policy message. In general, public opinion is most strongly influenced by the framing of the most recently received message, with the influence of earlier message frames diminishing over time [32].

From this study, there does not appear to be any value in including both inoculation and narrative components within the one advocacy message. It is possible that the combined message did not provide the same protective effect for anti-industry beliefs as the separate inoculation message due to it prompting increased counter-arguing of the advocacy message. The combined message was also around six minutes long and so those exposed to it may have been more easily distracted or more challenged by the task of processing all the differing message elements compared to those exposed to shorter, less cognitively demanding messages. While the literature suggests that longer text narratives are more effective than short text narratives, similar differences in persuasive impact based on message length are not apparent for audio-based narratives (i.e. the communication mode used in our study) [17]. Further, the influence of message length on the effectiveness of inoculation messages remains unknown, with no studies examining the role of this factor in resistance. Future experiments testing inoculation messages should consider designs that enable the effects of message length to be explored.

There was limited evidence that using inoculation and narrative strategies would provide additional benefit over and above standard pro-policy arguments in garnering public support for policy change when competing against strong industry opposition. However, while the standard advocacy message alone did not protect against the anti-policy message undoing otherwise unfavourable beliefs about the industry, the messages that also included the narrative or inoculation elements did protect against this. It may be that the inoculation and narrative sections were not prominent enough within the broader advocacy message, featuring a public health advocate conveying traditional arguments in support of the policy, to elicit a sufficiently strong persuasive advantage. Indeed, the manipulation check indicated that the narrative message did not successfully reduce counter-arguing of the advocacy message. Avoiding the undermining of negative beliefs about the industry is, however, an important end in itself, especially in light of well-funded efforts by the sugary drink and alcohol industries to cultivate a favourable public image and previous evidence that unfavourable beliefs about these industries are a strong predictor of support for a wide-range of health policies targeting these industries [33]. Thus, supplementing standard pro-policy arguments with inoculation or narrative messages may be critical if those messages can reduce favourable attitudes toward these industries.

A number of study limitations should be acknowledged. First, we recruited participants from two non-probability based online panels and achieved a low follow-up rate, with high non-response and dropout attrition also recorded at both time points. Thus the sample cannot be considered representative of the general Australian adult population. However, with the exception of age group (which was included as a covariate in the analyses), the sample profile was comparable across message conditions, suggesting that demographic characteristics did not confound the experiment. Second, to avoid priming and possible carryover/memory effects, we chose not to collect baseline measurements of policy support. Consequently, we could not examine whether participants’ prior policy attitudes moderated the magnitude or direction of the message effects. Third, in order to test the effects of advocacy messages as they would be implemented in the real world, there was substantial variation in message length across conditions which, due to collinearity, could not be accounted for in our analyses. Nonetheless, our finding that the longest message (i.e. Standard + I + N) was not the most effective suggests that additional content did not automatically increase message effectiveness. Fourth, no pilot testing of the advocacy messages or response measures was undertaken. The advocacy messages were, however, modelled upon those used in a previously published study that found effects of inoculation and narrative messages in the context of public health advocates seeking to implement policies to limit the marketing practices of the soda, tobacco and pharmaceutical industries [20]. Similarly, all our response measures were adapted from prior studies assessing the persuasive effects of health messages [19, 20, 29, 30]. Fifth, inoculation and narrative are theorised to have multiple but distinct pathways of influence. In an effort to manage respondent burden, we chose not to measure all of these mechanisms, relying on a single manipulation check for each message (perceived threat to freedom for inoculation; reduced counter-arguing for narrative). As noted above, exposure to the narrative conditions did not reduce counter-arguing of the advocacy message as theory would suggest. Nevertheless, exposure to the narrative condition (i.e. Standard + N) produced greater pro-policy attitudes at both t1 and t2 than the control group, suggesting the possibility that other (unmeasured) mechanisms may have been operating. Finally, the advocacy messages were audio-based, so readers should take caution in generalising the results to other message channels (e.g. print, video).

A strength of the present study was that we tested the advocacy messages across two distinct industries and two distinct policy tactics, thus providing stronger evidence regarding the potential utility of inoculation and/or narrative messages for public health advocates than if we had focused on a singular industry and policy tactic. Further, while examination of the covariate results in our regression analyses indicated that participant levels of policy support and anti-industry beliefs did significantly vary according to health policy assignment, we were importantly able to demonstrate, through interaction testing, that the advocacy messages produced comparable effects across policies.

## Conclusions

The findings from this research highlight the value of designing studies to replicate the real-world competitive messaging environment surrounding health policy issues. Specifically, it was demonstrated that important message effects can be overlooked when focusing solely on the immediate effects of exposure to advocacy messages. There is promising evidence that dissemination of advocacy messages that use inoculation or narrative strategies can make the public more resistant to future efforts at persuasion by industry groups. Future competitive framing research will help to further elucidate the particular conditions under which these effects may occur.

## Additional files

Additional file 1: Written transcripts of standard pro-policy arguments + inoculation + narrative (Standard + I + N) advocacy messages. This file contains the written transcripts of the Standard + I + N advocacy messages (marked up to identify the different message components). (PDF 165 kb)

Additional file 2: Written transcripts of industry anti-policy messages. This file contains the written transcripts of the industry anti-policy messages. (PDF 96 kb)

Additional file 3: Wording of survey items. This file contains the exact wording of the survey items and their corresponding response scales. (PDF 464 kb)

Additional file 4: Supplementary table. This file contains a supplementary table reporting the means and standard deviations for each outcome measure at Time 1 and Time 2 by message condition and health policy assignment. (PDF 87 kb)

## Abbreviations

H1: Hypothesis 1 – Inoculation and/or narrative messages will increase policy support and anti-industry beliefs relative to the control message immediately after exposure
H2: Hypothesis 2 – Inoculation messages will reduce the impact of a subsequent industry anti-policy message on policy support and anti-industry beliefs compared to the control message at a two-week follow-up
RQ1: Research question 1 – Does a narrative message (without inoculation components) offset the impact of a subsequent industry anti-policy message?
RQ2: Research question 2 – Do inoculation and/or narrative effects spill-over to other policies related to the same health issue?
RQ3: Research question 3 – Do inoculation and/or narrative messages produce stronger effects than a standard advocacy message?
Standard + I + N: Standard pro-policy arguments + inoculation + narrative
Standard + I: Standard pro-policy arguments + inoculation
Standard + N: Standard pro-policy arguments + narrative
Standard: Standard pro-policy arguments
t1: Time 1
t2: Time 2
