# Supplementary material for: Competing with big business: a randomised experiment testing the effects of messages to promote alcohol and sugary drink control policy
Source: BMC Public Health. 2017 Dec 28;17:945. doi: 10.1186/s12889-017-4972-6 (PMC5745776; doi:10.1186/s12889-017-4972-6)
Supplement: Supplementary file 1 — Written transcripts of standard pro-policy arguments + inoculation + narrative (Standard + I + N) advocacy messages. This file contains the written transcripts of the Standard + I + N advocacy messages (marked up to identify the different message components). (PDF 165 kb) [file 12889_2017_4972_MOESM1_ESM.pdf]

ADDITIONAL FILE 1:

Written transcripts of standard pro-policy arguments + inoculation + narrative (Standard+I+N) advocacy messages

1a: Transcript of radio segment for the Standard+I+N advocacy message promoting a 20% tax on sugary drinks

**Message Component**	**Script**
Interviewer	PRESENTER:<br>Welcome to the program. Today we are going to talk about why our waistlines are expanding.
Size and seriousness of health issue	PRESENTER:<br>Over the past 30 years, obesity rates in Australia have been increasing to the point that we are now one of the most overweight developed nations in the world. Almost two-thirds of adults and one in four children are overweight or obese.
Interviewer	PRESENTER:<br>Joining us to talk about this today is Claire Wallace from the Obesity Policy Coalition.<br><br>Welcome Claire. Why is this such a big problem?
Standard pro-policy argument	CLAIRE WALLACE:<br>You're right Kate, it is a big problem. Obesity has overtaken smoking as the number one risk factor for death and disease in Australia. Being above a healthy weight can cause diabetes, heart disease, and several types of cancer, and leads to other social and emotional problems such as depression and low self-esteem. Did you know that obesity also costs the country around 56 billion dollars each year in lost productivity, health care, and other social costs.
Interviewer	PRESENTER:<br>To hear more about how obesity is affecting Australian families, we spoke to 46 year-old mother of two from Queensland, Lisa Gilroy. Lisa recently learnt that her teenage daughter Cathy, who just turned 14, faced a range of serious health problems if she didn't lose weight.<br><br>Lisa, what was your reaction when your family GP told you this news?
Narrative	LISA GILROY:<br>I was shocked. While I had noticed that my daughter had gained some weight, I didn't realise it had reached a point where it could affect her health.
Interviewer	PRESENTER:<br>What did your GP recommend you do to help your daughter lose weight?
Narrative	LISA GILROY:<br>He suggested swapping sugary drinks such as soft drink, juice and cordial for water or low-fat milk instead. But we have never really had soft drink at home. I don't buy it, but it's available everywhere else she goes.
Interviewer	PRESENTER:<br>Does Cathy drink a lot of soft drink? Where else does she get it?

Narrative	LISA GILROY:<br>Soft drink and other sugary drinks are everywhere – at school, when she goes to the movies, or the local shopping centre. She’s a teenager with her own pocket money, and these drinks are relatively cheap. There’s also ads for soft drink and sports drinks all around her; they’re all over the telly, especially when she’s watching the football. Her sports club is even sponsored by a soft drink company, for goodness sake! Of course I encourage her to make healthy decisions, but it’s not realistic to compete against all those promotions showing young people partying and having fun.
Interviewer	PRESENTER:<br>Claire, I imagine that Lisa’s story is not unique.
Narrative	CLAIRE WALLACE:<br>No, it’s not – I hear similar stories everyday. It’s clear that Lisa is aware of the role she needs to play in protecting her daughter’s health. However, like many Australian parents, Lisa’s efforts to promote healthy habits and provide healthy food and drinks at home are being undermined by an environment that does not support healthy choices.
Interviewer	PRESENTER:<br>So Claire, what can we do to tackle this obesity crisis?
Standard pro-policy argument	CLAIRE WALLACE:<br>Well, we know that one of the factors driving this obesity problem is the consumption of sugary drinks. Sugary drinks, such as soft drinks – the non-diet ones –, sports drinks, energy drinks, and cordials, are especially high in kilojoules and are a clear cause of overweight and obesity. Drinking even one can of soft drink a day is enough to make a person gain more than 5 kilograms of excess weight in a year.<br><br>That’s why the Obesity Policy Coalition, along with other leading public health groups internationally and in Australia, are calling for a 20% tax on sugary drinks.
Interviewer	PRESENTER:<br>The soft drink industry strongly opposes your plan to tax sugary drinks, don’t they?
Inoculation	CLAIRE WALLACE:<br>Well, of course they do. These companies spend millions each year marketing products that have no nutritional value. They will say and do almost anything to protect their profits, and they do it at the expense of Australia’s health. Behind the scenes, they are relentlessly pressuring the government to oppose any policies they see as a threat to their bottom line.
Interviewer	PRESENTER:<br>Soft drink companies claim they are being unfairly targeted. How do you respond to that?
Inoculation	CLAIRE WALLACE:<br>Soft drink companies will try to convince us that a tax is arbitrary because it singles out sugary drinks and does not affect things like fast food, chocolate and lollies. They will say that these taxes are just a quick way for politicians to fill budget holes. They will call them “food taxes” to try to confuse people, and they will make misleading claims that Australians are drinking fewer soft drinks than in the past.

Interviewer	PRESENTER:<br>So why are public health groups focusing specifically on sugary drinks?
Standard pro-policy argument + inoculation	CLAIRE WALLACE:<br>The fact is that sugary drinks need to be singled out because they are quite unique – they have no nutritional value, just enormous amounts of sugar. Did you know that a 600ml bottle of soft drink contains up to 16 teaspoons of sugar? That's more than double the daily amount recommended by the World Health Organization! We know that price is an important factor in what people consume, particularly for young people who drink the most sugary drinks. Nobody is telling anyone what to drink. But, by increasing the price of sugary drinks, many people will choose differently.
Interviewer	PRESENTER:<br>But, is a tax on sugary drinks going to fix Australia's obesity problem?
Standard pro-policy argument	CLAIRE WALLACE:<br>A tax on sugary drinks is one important part of the solution, but it's not the only solution. The obesity problem needs to be tackled on many different fronts. Increasing the price of sugary drinks will make these drinks less attractive, and combined with other measures, it will help reduce how much people drink. This is important, as we know the more sugary drinks people consume the greater the risk they will become overweight and suffer from chronic diseases. A tax on sugary drinks will also provide new money to fund school and community education programs to help reduce childhood obesity. This money will also go towards reducing the cost of healthy food in supermarkets and green grocers for low-income families and students.
Interviewer	PRESENTER:<br>It's a measure supported by Lisa Gilroy, as she tries to get her teenage daughter to lose weight.
Narrative	LISA GILROY:<br>As her mother, I know that I have an important role to play in helping my daughter make healthy choices. But any policy that makes these drinks less affordable – like the new tax that's being proposed – would be a real help.

1b: Transcript of radio segment for the Standard+I+N advocacy message promoting the removal of sugary drink sponsorship from sport

**Message Component**	**Script**
Interviewer	PRESENTER:<br>Welcome to the program. Today we are going to talk about why our waistlines are expanding.
Size and seriousness of health issue	PRESENTER:<br>Over the past 30 years, obesity rates in Australia have been increasing to the point that we are now one of the most overweight developed nations in the world. Almost two-thirds of adults and one in four children are overweight or obese.
Interviewer	PRESENTER:<br>Joining us to talk about this today is Claire Wallace from the Obesity Policy Coalition.<br><br>Welcome Claire. Why is this such a big problem?
Standard pro-policy argument	CLAIRE WALLACE:<br>You're right Kate, it is a big problem. Obesity has overtaken smoking as the number one risk factor for death and disease in Australia. Being above a healthy weight can cause diabetes, heart disease, and several types of cancer, and leads to other social and emotional problems such as depression and low self-esteem. Did you know that obesity also costs the country around 56 billion dollars each year in lost productivity, health care, and other social costs.
Interviewer	PRESENTER:<br>To hear more about how obesity is affecting Australian families, we spoke to 46 year-old mother of two from Queensland, Lisa Gilroy. Lisa recently learnt that her teenage daughter Cathy, who just turned 14, faced a range of serious health problems if she didn't lose weight.<br><br>Lisa, what was your reaction when your family GP told you this news?
Narrative	LISA GILROY:<br>I was shocked. While I had noticed that my daughter had gained some weight, I didn't realise it had reached a point where it could affect her health.
Interviewer	PRESENTER:<br>What did your GP recommend you do to help your daughter lose weight?
Narrative	LISA GILROY:<br>He suggested swapping sugary drinks such as soft drink, juice and cordial for water or low-fat milk instead. But we have never really had soft drink at home. I don't buy it, but it's available everywhere else she goes.
Interviewer	PRESENTER:<br>Does Cathy drink a lot of soft drink? Where else does she get it?

Narrative	LISA GILROY:<br>Soft drink and other sugary drinks are everywhere – at school, when she goes to the movies, or the local shopping centre. She's a teenager with her own pocket money, and these drinks are relatively cheap. There's also ads for soft drink and sports drinks all around her; they're all over the telly, especially when she's watching the football. Her sports club is even sponsored by a soft drink company, for goodness sake! Of course I encourage her to make healthy decisions, but it's not realistic to compete against all those promotions showing young people partying and having fun.
Interviewer	PRESENTER:<br>Claire, I imagine that Lisa's story is not unique.
Narrative	CLAIRE WALLACE:<br>No, it's not – I hear similar stories everyday. It's clear that Lisa is aware of the role she needs to play in protecting her daughter's health. However, like many Australian parents, Lisa's efforts to promote healthy habits and provide healthy food and drinks at home are being undermined by an environment that does not support healthy choices.
Interviewer	PRESENTER:<br>So Claire, what can we do to tackle this obesity crisis?
Standard pro-policy argument	CLAIRE WALLACE:<br>Well, we know that one of the factors driving this obesity problem is the consumption of sugary drinks. Sugary drinks, such as soft drinks – the non-diet ones –, sports drinks, energy drinks, and cordials, are especially high in kilojoules and are a clear cause of overweight and obesity. Drinking even one can of soft drink a day is enough to make a person gain more than 5 kilograms of excess weight in a year.<br><br>That's why the Obesity Policy Coalition, along with other leading public health groups internationally and in Australia, are calling for the removal of sugary drink sponsorship from sport.
Interviewer	PRESENTER:<br>The soft drink industry strongly opposes your plan to ban sugary drink sponsorship of sport, don't they?
Inoculation	CLAIRE WALLACE:<br>Well, of course they do. These companies spend millions each year using Australian sporting heroes to market products that have no nutritional value. They will say and do almost anything to protect their profits, and they do it at the expense of Australia's health. Behind the scenes, they are relentlessly pressuring the government to oppose any policies they see as a threat to their bottom line.
Interviewer	PRESENTER:<br>Soft drink companies claim they are being unfairly targeted. How do you respond to that?
Inoculation	CLAIRE WALLACE:<br>Soft drink companies will try to convince us that this restriction is arbitrary because it singles out sugary drinks and does not affect sponsorship by companies marketing things like fast food, chocolate and lollies. They will say that these restrictions are the government trying to control the activities of legal businesses. They'll even say they can be trusted to regulate themselves, and they will make misleading claims that Australians are drinking fewer soft drinks than in the past.

Interviewer	PRESENTER:<br>So why are public health groups focusing specifically on sugary drinks?
Standard pro-policy argument + inoculation	CLAIRE WALLACE:<br>The fact is that sugary drinks need to be singled out because they are quite unique – they have no nutritional value, just enormous amounts of sugar. Did you know that a 600ml bottle of soft drink contains up to 16 teaspoons of sugar? That's more than double the daily amount recommended by the World Health Organization! Soft drink companies say they don't target young people, but that's exactly what they are doing when they use sporting teams and sporting heroes to market their products. Their only goal is to sell as much soft drink and make as much money as possible. So why should we trust *them* to stop young people from consuming their products?
Interviewer	PRESENTER:<br>But, is removing sugary drink sponsorship from sport going to fix Australia's obesity problem?
Standard pro-policy argument	CLAIRE WALLACE:<br>Removing sugary drink sponsorship from sport is one important part of the solution, but it's not the only solution. The obesity problem needs to be tackled on many different fronts. Restricting where, when and how sugary drinks can be marketed will make these drinks less attractive to young people. We know it's important to reduce young people's exposure to this marketing because it influences how much they consume, and the more kids drink sugary drinks the greater the risk they will become overweight and suffer from chronic diseases later in life. Removing sugary drink sponsorship from sport will reduce the unhealthy messages that young people are exposed to when they watch their favourite sporting teams. It will also help parents encourage their kids to make healthier choices.
Interviewer	PRESENTER:<br>It's a measure supported by Lisa Gilroy, as she tries to get her teenage daughter to lose weight.
Narrative	LISA GILROY:<br>As her mother, I know that I have an important role to play in helping my daughter make healthy choices. But any policy that makes these drinks less appealing – like the sport sponsorship bans that are being proposed – would help to make it more of a level playing field.

1c: Transcript of radio segment for the Standard+I+N advocacy message promoting a volume-based tax on alcohol

Message Component	Script
Interviewer	PRESENTER:<br>Welcome to the program. Today we are going to talk about why alcohol is causing so much harm in our community.
Size and seriousness of health issue	PRESENTER:<br>Over the past 20 years, Australia's alcohol consumption has remained high – currently we rank 19th out of 194 countries in per capita alcohol consumption. More than one in five Australians drinks in a way that puts their health and safety at risk, and more than a quarter of Australian youth start drinking before their 18th birthday.
Interviewer	PRESENTER:<br>Joining us to talk about this today is Claire Wallace from the Alcohol Policy Coalition.<br><br>Welcome Claire. Why is this such a big problem?
Standard pro-policy argument	CLAIRE WALLACE:<br>You're right Kate, it is a big problem. Alcohol is a major risk factor for death and disease in Australia. Harmful alcohol consumption can cause liver disease, heart disease and several types of cancer, and it increases the risk of accidents, injury and violence for both the drinker and others. Did you know that alcohol misuse also costs the country around 36 billion dollars each year in lost productivity, health care, and other social costs.
Interviewer	PRESENTER:<br>To hear more about how alcohol is affecting Australian families, we spoke to 46 year-old mother of two from Queensland, Lisa Gilroy. Lisa recently learnt that her teenage daughter Cathy, who just turned 14, has started drinking at parties with her friends.<br><br>Lisa, what was your reaction when you found this out?
Narrative	LISA GILROY:<br>I was shocked. While I knew my daughter was reaching the age when young people start to experiment with alcohol, I didn't think *she* would be drinking just yet. I did some research on the 'net' for ideas about what we could say or do to try to keep her from drinking and after that we sat down and talked to her…
Interviewer	PRESENTER:<br>What did you say to try to keep your daughter from drinking alcohol?
Narrative	LISA GILROY:<br>We talked to her about the dangers of drinking and some strategies to deal with peer pressure to drink. Also, while my husband and I aren't teetotallers, we've always been mindful of setting a good example when it comes to alcohol. We never have more than 1 or 2 drinks in front of her and have never given her alcohol.
Interviewer	PRESENTER:<br>Does Cathy drink a lot of alcohol? Where does she get it?

Narrative	LISA GILROY:<br>Alcohol is available everywhere – there's a bottle shop on every corner, and you can get it at the local supermarket and at every sporting event. She's a teenager with her own pocket money, and her friends have older siblings, so she seems to be able to find a way to get hold of these drinks. There's also ads for beer and wine all around her: they're all over the telly, especially when she's watching the football. Her sports club even has big signs for those fruit flavoured alcopops, for goodness sake! Of course I encourage her not to drink, but it's not realistic to compete against all those promotions showing young people partying and having fun.
Interviewer	PRESENTER:<br>Claire, I imagine that Lisa's story is not unique.
Narrative	CLAIRE WALLACE:<br>No, it's not – I hear similar stories everyday. It's clear that Lisa is aware of the role she needs to play in protecting her daughter's health. However, like many Australian parents, Lisa's efforts to educate her daughter about the dangers of alcohol and to model sensible drinking behaviours at home are being undermined by an environment that makes drinking seem like the normal thing to do.
Interviewer	PRESENTER:<br>So Claire, what can we do to tackle this problem with underage drinking?
Standard pro-policy argument	CLAIRE WALLACE:<br>Well, we know that one of the factors driving this alcohol problem is the ready access people have to affordable alcohol, including products that have very high alcohol content at relatively low prices. For example, under the current system, it's possible to get a bottle of sweet fizzy wine (like Passion Pop) that contains more than 5 standard drinks for less than $5. That's a lot of alcohol if consumed in one sitting, especially by a teenager. Young people are particularly price sensitive which means that the cost of alcohol has a big influence on how much they drink and when they start to drink.<br><br>That's why the Alcohol Policy Coalition, along with other leading public health groups internationally and in Australia, are calling for a volume-based tax on alcohol products. This means that all alcohol products would be taxed according to their alcohol content.
Interviewer	PRESENTER:<br>The alcohol industry strongly opposes your plan to increase the price of alcohol, don't they?
Inoculation	CLAIRE WALLACE:<br>Well, of course they do. These companies spend millions each year marketing their products to young people. They will say and do almost anything to protect their profits, and they do it at the expense of Australia's health. Behind the scenes, they are relentlessly pressuring the government to oppose any policies they see as a threat to their bottom line.
Interviewer	PRESENTER:<br>Alcohol companies claim that these taxes are ineffective and unnecessary. How do you respond to that?

Inoculation	CLAIRE WALLACE:<br>Alcohol companies will try to convince us that a volume-based tax won't work because those who are dependent on alcohol will continue to drink regardless, penalising responsible drinkers without deterring heavy drinkers. They will say that these taxes are just a quick way for politicians to fill budget holes, and they'll make misleading claims that higher alcohol prices will hurt the poor more than the rich.
Interviewer	PRESENTER:<br>So why are public health groups calling for a change to the current alcohol taxation system?
Standard pro-policy argument + inoculation	CLAIRE WALLACE:<br>The fact is that alcohol is no ordinary product – it's responsible for major harms to drinkers, their families, and the wider community. Did you know that in 2013, nearly 5 million Australians were the victim of an alcohol-related incident such as verbal or physical abuse? That's more than the entire population of Sydney! We know that price is an important factor in how much people drink, particularly for young people. Nobody is telling anyone that they can't drink. But, by changing the tax system so that products are taxed based on their alcohol content, many people will be encouraged to drink less.
Interviewer	PRESENTER:<br>But, are these alcohol tax changes going to fix Australia's drinking problem?
Standard pro-policy argument	CLAIRE WALLACE:<br>A volume-based tax on alcohol is one important part of the solution, but it's not the only solution. The alcohol problem needs to be tackled on many different fronts. In general, this tax will make products that have a higher alcohol content more expensive, which will give people an incentive to drink lower alcohol products. Combined with other measures, it will help reduce how much people drink, which will reduce their risk of serious health harms, chronic disease and injuries. This tax will also be used to provide more money to fund school and community education programs, as well as alcohol treatment programs and better health care for the whole community.
Interviewer	PRESENTER:<br>It's a measure supported by Lisa Gilroy, as she tries to protect her daughter from the dangers associated with underage drinking.
Narrative	LISA GILROY:<br>As her mother, I know that I have an important role to play in helping my daughter make responsible choices. But any policy that makes alcohol less affordable – like the tax changes that are being proposed – would be a real help.

1d: Transcript of radio segment for the Standard+I+N advocacy message promoting the removal of alcohol sponsorship from sport

**Message Component**	**Script**
Interviewer	PRESENTER:<br>Welcome to the program. Today we are going to talk about why alcohol is causing so much harm in our community.
Size and seriousness of health issue	PRESENTER:<br>Over the past 20 years, Australia's alcohol consumption has remained high – currently we rank 19th out of 194 countries in per capita alcohol consumption. More than one in five Australians drinks in a way that puts their health and safety at risk, and more than a quarter of Australian youth start drinking before their 18th birthday.
Interviewer	PRESENTER:<br>Joining us to talk about this today is Claire Wallace from the Alcohol Policy Coalition.<br><br>Welcome Claire. Why is this such a big problem?
Standard pro-policy argument	CLAIRE WALLACE:<br>You're right Kate, it is a big problem. Alcohol is a major risk factor for death and disease in Australia. Harmful alcohol consumption can cause liver disease, heart disease and several types of cancer, and it increases the risk of accidents, injury and violence for both the drinker and others. Did you know that alcohol misuse also costs the country around 36 billion dollars each year in lost productivity, health care, and other social costs.
Interviewer	PRESENTER:<br>To hear more about how alcohol is affecting Australian families, we spoke to 46 year-old mother of two from Queensland, Lisa Gilroy. Lisa recently learnt that her teenage daughter Cathy, who just turned 14, has started drinking at parties with her friends.<br><br>Lisa, what was your reaction when you found this out?
Narrative	LISA GILROY:<br>I was shocked. While I knew my daughter was reaching the age when young people start to experiment with alcohol, I didn't think *she* would be drinking just yet. I did some research on the 'net' for ideas about what we could say or do to try to keep her from drinking and after that we sat down and talked to her…
Interviewer	PRESENTER:<br>What did you say to try to keep your daughter from drinking alcohol?
Narrative	LISA GILROY:<br>We talked to her about the dangers of drinking and some strategies to deal with peer pressure to drink. Also, while my husband and I aren't teetotallers, we've always been mindful of setting a good example when it comes to alcohol. We never have more than 1 or 2 drinks in front of her and have never given her alcohol.
Interviewer	PRESENTER:<br>Does Cathy drink a lot of alcohol? Where does she get it?

Narrative	LISA GILROY:<br>Alcohol is available everywhere – there's a bottle shop on every corner, and you can get it at the local supermarket and at every sporting event. She's a teenager with her own pocket money, and her friends have older siblings, so she seems to be able to find a way to get hold of these drinks. There's also ads for beer and wine all around her: they're all over the telly, especially when she's watching the football. Her sports club even has big signs for those fruit flavoured alcopops, for goodness sake! Of course I encourage her not to drink, but it's not realistic to compete against all those promotions showing young people partying and having fun.
Interviewer	PRESENTER:<br>Claire, I imagine that Lisa's story is not unique.
Narrative	CLAIRE WALLACE:<br>No, it's not – I hear similar stories everyday. It's clear that Lisa is aware of the role she needs to play in protecting her daughter's health. However, like many Australian parents, Lisa's efforts to educate her daughter about the dangers of alcohol and to model sensible drinking behaviours at home are being undermined by an environment that makes drinking seem like the normal thing to do.
Interviewer	PRESENTER:<br>So Claire, what can we do to tackle this problem with underage drinking?
Standard pro-policy argument	CLAIRE WALLACE:<br>Well, we know that one of the factors driving this problem is that young people today are exposed to more advertising and promotion for alcohol than ever before: it's on their TV and mobile phone screens and on billboards around their schools, and so many of our sporting and cultural events in Australia are sponsored by alcohol companies. Young people who are exposed to alcohol marketing are more likely to start drinking at a younger age, and to drink more alcohol once they do start.<br><br>That's why the Alcohol Policy Coalition, along with other leading public health groups internationally and in Australia, are calling for the removal of alcohol sponsorship from sport.
Interviewer	PRESENTER:<br>The alcohol industry strongly opposes your plan to ban alcohol sponsorship of sport, don't they?
Inoculation	CLAIRE WALLACE:<br>Well, of course they do. These companies spend millions each year using Australian sporting heroes to market their products to young people. They will say and do almost anything to protect their profits, and they do it at the expense of Australia's health. Behind the scenes, they are relentlessly pressuring the government to oppose any policies they see as a threat to their bottom line.
Interviewer	PRESENTER:<br>Alcohol companies claim these bans are ineffective and unnecessary. How do you respond to that?

Inoculation	CLAIRE WALLACE:<br>Alcohol companies will try to convince us that peer pressure and the drinking habits of their parents are what leads to underage drinking, and that advertising alcohol through sporting sponsorships has nothing to do with it. They will say that these restrictions are the government trying to control the activities of legal businesses. They'll even say they can be trusted to regulate themselves, and they will make misleading claims that young Australians are drinking less than in the past.
Interviewer	PRESENTER:<br>So why are public health groups so concerned about alcohol sponsorship of sport?
Standard pro-policy argument + inoculation	CLAIRE WALLACE:<br>The fact is that alcohol is no ordinary product – it's responsible for major harms to drinkers, their families, and the wider community. Did you know that in 2013, nearly 5 million Australians were the victim of an alcohol-related incident such as verbal or physical abuse? That's more than the entire population of Sydney! Alcohol companies say they don't target young people, but that's exactly what they are doing when they use sporting teams and sporting heroes to market their products. Their only goal is to sell as much alcohol and make as much money as possible. So why should we trust *them* to stop encouraging young people to drink.
Interviewer	PRESENTER:<br>But, is removing alcohol sponsorship from sport going to fix Australia's drinking problem?
Standard pro-policy argument	CLAIRE WALLACE:<br>Removing alcohol sponsorship from sport is one important part of the solution, but it's not the only solution. The alcohol problem needs to be tackled on many different fronts. Restricting where, when and how alcohol can be marketed will make drinking less attractive to young people. We know that it's important to reduce young people's exposure to alcohol marketing because it influences their drinking habits, and the earlier kids start drinking the greater the risk they will suffer from alcohol-related harms both immediately and later in life. Removing alcohol sponsorship from sport will reduce the pro-drinking messages that young people are exposed to when they watch their favourite sporting teams. It will also help parents to take better control over the types of messages their kids see about drinking alcohol.
Interviewer	PRESENTER:<br>It's a measure supported by Lisa Gilroy, as she tries to protect her daughter from the dangers associated with underage drinking.
Narrative	LISA GILROY:<br>As her mother, I know that I have an important role to play in helping my daughter make responsible choices. But any policy that makes alcohol less appealing – like the sport sponsorship bans that are being proposed – would help to make it more of a level playing field.
