# Supplementary material for: Competing with big business: a randomised experiment testing the effects of messages to promote alcohol and sugary drink control policy
Source: BMC Public Health. 2017 Dec 28;17:945. doi: 10.1186/s12889-017-4972-6 (PMC5745776; doi:10.1186/s12889-017-4972-6)
Supplement: Supplementary file 2 — Written transcripts of industry anti-policy messages. This file contains the written transcripts of the industry anti-policy messages. (PDF 96 kb) [file 12889_2017_4972_MOESM2_ESM.pdf]

ADDITIONAL FILE 2:
Written transcripts of industry anti-policy messages

2a: Transcript of radio segment for the industry anti-policy message regarding a 20% tax on sugary drinks

**Script**
PRESENTER:<br>Leading public health groups in Australia, including the Obesity Policy Coalition, are calling for a 20% tax on sugary drinks to help tackle the nation’s growing obesity issue.<br><br>We’re now joined by Michael Harris, a spokesperson from the soft drink industry, to get his thoughts on this policy proposal.<br><br>Welcome Michael. Tell us, do you believe that a tax on sugary drinks has merit?
MICHAEL HARRIS:<br>The headlines are hard to ignore, Australians need to lose weight. However, this serious and complex problem cannot be solved by focusing on a small piece of the total diet. Science shows that obesity is caused by an imbalance between the kilojoules we consume through food and drinks and those we burn through daily activities and exercise. To single out soft drink as some kind of unique contributor to obesity is arbitrary and misguided. In Australia, less than 2 per cent of the daily intake of kilojoules for adults and children comes from soft drinks. In fact, research shows that sugar consumption from soft drinks in Australia has decreased in recent years, while obesity rates continue to increase. We have to look at the bigger picture.
PRESENTER:<br>Well, what can we do to reduce obesity?
MICHAEL HARRIS:<br>If we want a healthier country, we need better education about exercise and balanced diets, not a nanny-state tax on the foods that we eat and things that we drink. It’s never the government’s job to decide what you should eat or drink – people can decide for themselves.
PRESENTER:<br>So, what role can the soft drink industry play in reversing the current obesity trend?
MICHAEL HARRIS:<br>We are making a strong effort to ensure that we’re part of the solution. The industry produces a broad range of products to suit everybody’s lifestyle, taste and kilojoule needs. The fact that two of the three biggest selling soft drinks in Australia are now zero or low kilojoule shows that companies are taking action through innovation and reformulation to provide consumers with variety and choices consistent with healthy lifestyles.<br><br>Taxing one particular nutrient, or a particular food or drink ignores the bigger problem, and won’t help Australians address their expanding waistlines. It will only line government coffers, while hitting Australian families where it hurts most – their hip pockets.

2b: Transcript of radio segment for the industry anti-policy message regarding the removal of sugary drink sponsorship from sport

Script
PRESENTER:<br>Leading public health groups in Australia, including the Obesity Policy Coalition, are calling for the removal of sugary drink sponsorship from sport to help tackle the nation's growing obesity issue.<br><br>We're now joined by Michael Harris, a spokesperson from the soft drink industry, to get his thoughts on this policy proposal.<br><br>Welcome Michael. Tell us, do you believe that a ban on sugary drink sponsorship of sport has merit?
MICHAEL HARRIS:<br>The headlines are hard to ignore, Australians need to lose weight. However, banning soft drink sponsorship of sport is a simplistic approach to a complex societal issue. Science shows that obesity is caused by an imbalance between the kilojoules we consume through food and drinks and those we burn through daily activities and exercise. To single out soft drink as some kind of unique contributor to obesity is arbitrary and misguided. In Australia, less than 2 per cent of the daily intake of kilojoules for adults and children comes from soft drinks. The idea that sport sponsorship by soft drink companies is driving consumption and weight gain is not backed by facts. Research shows that sugar consumption from soft drinks in Australia has decreased in recent years, while obesity rates continue to increase. We have to look at the bigger picture.
PRESENTER:<br>Well, what can we do to reduce obesity?
MICHAEL HARRIS:<br>If we want a healthier country, we need better education about exercise and balanced diets, not a nanny-state ban on sport sponsorship that prevents legitimate companies, who employ thousands of hard-working Australians, from promoting their products.
PRESENTER:<br>So, what role can the soft drink industry play in reversing the current obesity trend?
MICHAEL HARRIS:<br>We are making a strong effort to ensure that we're part of the solution. Australia's beverage companies understand the responsibilities associated with marketing to young people. That's why we have, for a long time, voluntarily adopted a policy not to advertise soft drinks to children under 12 years of age. We use marketing, such as sponsorship of sport, to influence brand choices, not to encourage consumption. We're also proactive in promoting healthy choices such as juice and water for kids and their parents. These responsible practices recognise the central role that parents must play in making choices for their own children.<br><br>Banning soft drink sponsorship from sport ignores the bigger problem, and won't help Australians address their expanding waistlines. It will only take much needed money away from sporting competitions and clubs who promote healthy, active lifestyles and who would not survive without the valuable sponsorship and support of these companies.

2c: Transcript of radio segment for the industry anti-policy message regarding a volume-based tax on alcohol

**Script**
PRESENTER:<br>Leading public health groups in Australia, including the Alcohol Policy Coalition, are calling for a volume-based tax on alcohol products so that all drinks are taxed according to their alcohol content. These efforts are intended to help tackle the nation's drinking problem.<br><br>We're now joined by Michael Harris, a spokesperson from the alcohol industry, to get his thoughts on this policy proposal.<br><br>Welcome Michael. Tell us, do you believe that a volume-based tax system for alcohol has merit?
MICHAEL HARRIS:<br>The headlines are hard to ignore, too many Australians are affected by alcohol-related harms. However, this serious and complex problem cannot be solved by increasing taxes on alcohol. Research shows that demand for alcohol among those who drink in dangerous ways is not affected by price, meaning this new tax system would not necessarily bring about a drop in risky drinking levels. Using tax as a device to prevent alcohol abuse is misguided, and will only result in responsible adult drinkers paying an unnecessarily high price for alcohol. These taxes will place the greatest burden on society's poorest citizens – those with the least means to cope with price increases. We have to look at the bigger picture.
PRESENTER:<br>Well, what can we do to reduce alcohol-related harms?
MICHAEL HARRIS:<br>Alcohol policy should be based on establishing a drinking culture that maximises enjoyment and benefits of moderate consumption while tackling the harms caused by alcohol abuse. For the minority of people who misuse alcohol, we support greater education, and where necessary, targeted interventions, not a nanny-state tax increase on products that the majority of people consume responsibly.
PRESENTER:<br>So, what role can the alcohol industry play in promoting a safer drinking culture in Australia?
MICHAEL HARRIS:<br>We are making a strong effort to ensure that we're part of the solution. The industry produces a broad range of alcoholic products, including low and mid-strength options, to suit everybody's lifestyle and taste preferences. We also actively seek to reduce underage drinking through encouraging enforcement of government regulation of under-age purchase and consumption.<br><br>Increasing the price of alcohol ignores the bigger problem, and won't help protect Australians from alcohol-related harms. It will only line government coffers, while hitting hard-working Australians where it hurts most – their hip pockets.

2d: Transcript of radio segment for the industry anti-policy message regarding the removal of alcohol sponsorship from sport

Script
PRESENTER:<br>Leading public health groups in Australia, including the Alcohol Policy Coalition, are calling for the removal of alcohol sponsorship from sport to help tackle the nation's drinking problem.<br><br>We're now joined by Michael Harris, a spokesperson from the alcohol industry, to get his thoughts on this policy proposal.<br><br>Welcome Michael. Tell us, do you believe that a ban on alcohol sponsorship of sport has merit?
MICHAEL HARRIS:<br>The headlines are hard to ignore, too many Australians are affected by alcohol-related harms. However, banning alcohol sponsorship of sport is a simplistic approach to a complex societal issue. Research tells us there are a wide variety of social, environmental, and personal reasons why people engage in risky drinking behaviours. To single out sport sponsorship as some kind of direct cause of alcohol misuse and underage drinking is misguided. The primary influences on young people's attitudes to alcohol are their parents and peers; not a logo on a footballer's jumper. The idea that sport sponsorship is driving consumption and underage drinking is not backed by facts. Overall, consumption is down and there are fewer under-aged drinkers. We have to look at the bigger picture.
PRESENTER:<br>Well, what can we do to reduce alcohol-related harms?
MICHAEL HARRIS:<br>Alcohol policy should be based on establishing a drinking culture that maximises enjoyment and benefits of moderate consumption while tackling the harms caused by alcohol abuse. For the minority of people who misuse alcohol, we support greater education, and where necessary, targeted interventions, not a nanny-state ban on sport sponsorship that prevents legitimate companies, who employ thousands of hard-working Australians, from promoting their products to adults.
PRESENTER:<br>So, what role can the alcohol industry play in promoting a safer drinking culture in Australia?
MICHAEL HARRIS:<br>We are making a strong effort to ensure that we're part of the solution. Australia's alcohol companies understand the importance of ensuring their marketing is directed only at those over the legal age and is carried out in a socially responsible manner. That's why we have a strict, comprehensive industry code that complements existing legal frameworks to provide additional consumer protection. We use marketing, such as sponsorship of sport, not to encourage underage drinking, but to influence the brand choices of *adult* drinkers who have the right to make up their own mind about what they drink.<br><br>Banning alcohol sponsorship from sport ignores the bigger problem, and won't help protect Australians from alcohol-related harms. It will only take much needed money away from sporting competitions and clubs who promote healthy, active lifestyles and who would not survive without the valuable sponsorship and support of these companies.
