# Supplementary material for: Competing with big business: a randomised experiment testing the effects of messages to promote alcohol and sugary drink control policy
Source: BMC Public Health. 2017 Dec 28;17:945. doi: 10.1186/s12889-017-4972-6 (PMC5745776; doi:10.1186/s12889-017-4972-6)
Supplement: Supplementary file 3 — Wording of survey items. This file contains the exact wording of the survey items and their corresponding response scales. (PDF 464 kb) [file 12889_2017_4972_MOESM3_ESM.pdf]

ADDITIONAL FILE 3: Wording of survey items

Measure	Question		Response scale
	**Participants assigned to a sugary drink policy area**	**Participants assigned to an alcohol policy area**	
Policy support<br><br>[Asked of all participants at both time points]	There are many different ways that we as a society could deal with the issue of obesity. Which of these strategies would you support and which would you oppose?<br>• A 20 percent tax on sugary drinks<br>• Removing sugary drink sponsorship from sport<br>• Requiring large and prominently placed health warning labels on sugary drinks	There are many different ways that we as a society could deal with the issue of alcohol-related harm. Which of these strategies would you support and which would you oppose?<br>• A volume-based tax on alcohol products so that all drinks are taxed according to their alcohol content<br>• Removing alcohol sponsorship from sport<br>• Requiring large and prominently placed health warning labels on alcohol containers	1 – Strongly oppose<br>2 – Oppose<br>3 – Somewhat oppose<br>4 – Neither oppose nor support<br>5 – Somewhat support<br>6 – Support<br>7 – Strongly support
Anti-industry beliefs<br><br>[Asked of all participants at both time points]	Please read the following statements about soft drink companies. For each one, please indicate how much you agree or disagree with the statement.<br>• Soft drink companies deny that sugary drinks cause obesity<br>• Soft drink companies only care about making a lot of money<br>• Soft drink companies try to get young people to drink sugary drinks	Please read the following statements about alcohol companies. For each one, please indicate how much you agree or disagree with the statement.<br>• Alcohol companies deny they market their products to young people<br>• Alcohol companies only care about making a lot of money<br>• Alcohol companies try to get young people to drink alcohol	1 – Strongly disagree<br>2 – Disagree<br>3 – Somewhat disagree<br>4 – Neither disagree nor agree<br>5 – Somewhat agree<br>6 – Agree<br>7 – Strongly agree

Measure	Question		Response scale
	**Participants assigned to a sugary drink policy area**	**Participants assigned to an alcohol policy area**	
Perceived threat to freedom<br><br>[Only asked of participants assigned to one of the four advocacy messages at Time 1]	Please indicate how much you agree or disagree with each of the following statements.<br><br>When thinking about how we as a society deal with the problem of obesity, soft drink companies will:<br>• Try to tell me what to think<br>• Try to make up my mind for me<br>• Try to manipulate my thoughts<br>• Try to pressure me	Please indicate how much you agree or disagree with each of the following statements.<br><br>When thinking about how we as a society should deal with the problem of alcohol-related harm, alcohol companies will:<br>• Try to tell me what to think<br>• Try to make up my mind for me<br>• Try to manipulate my thoughts<br>• Try to pressure me	1 – Strongly disagree<br>2 – Disagree<br>3 – Somewhat disagree<br>4 – Neither disagree nor agree<br>5 – Somewhat agree<br>6 – Agree<br>7 – Strongly agree
Counter-arguing<br><br>[Only asked of participants assigned to one of the four advocacy messages at Time 1]	In the message you just heard, the host of the radio program spoke to one or two people to get their views on the obesity issue. Please think about the points that were made by these people during the interview, and then indicate how much you agree or disagree with each of the following statements.<br>• I found myself agreeing with the interviewee/s points<br>• I found myself disagreeing with the interviewee/s points<br>• I thought of a lot of arguments against what the interviewee/s were saying<br>• I accepted a lot of the arguments the interviewee/s offered	In the message you just heard, the host of the radio program spoke to one or two people to get their views on the alcohol issue. Please think about the points that were made by these people during the interview, and then indicate how much you agree or disagree with each of the following statements.<br>• I found myself agreeing with the interviewee/s points<br>• I found myself disagreeing with the interviewee/s points<br>• I thought of a lot of arguments against what the interviewee/s were saying<br>• I accepted a lot of the arguments the interviewee/s offered	1 – Strongly disagree<br>2 – Disagree<br>3 – Somewhat disagree<br>4 – Neither disagree nor agree<br>5 – Somewhat agree<br>6 – Agree<br>7 – Strongly agree
