# Supplementary material for: Competing with big business: a randomised experiment testing the effects of messages to promote alcohol and sugary drink control policy
Source: BMC Public Health. 2017 Dec 28;17:945. doi: 10.1186/s12889-017-4972-6 (PMC5745776; doi:10.1186/s12889-017-4972-6)
Supplement: Supplementary file 4 — Supplementary table. This file contains a supplementary table reporting the means and standard deviations for each outcome measure at Time 1 and Time 2 by message condition and health policy assignment. (PDF 87 kb) [file 12889_2017_4972_MOESM4_ESM.pdf]

ADDITIONAL FILE 4: Supplementary table

Supplementary Table: Means and standard deviations for each outcome measure at Time 1 (n=6,000) and Time 2 (n=3,285) by message condition and health policy assignment

	Target policy support[a]		Anti-industry beliefs[b]		Average non-target policy support[c]	
	**Time 1**	**Time 2**	**Time 1**	**Time 2**	**Time 1**	**Time 2**
	M (sd)	M (sd)	M (sd)	M (sd)	M (sd)	M (sd)
Message condition						
Control	4.78 (1.86)	3.54 (1.91)	5.47 (1.04)	4.97 (1.17)	5.09 (1.41)	4.60 (1.53)
Standard pro-policy arguments (Standard)	4.89 (1.85)	3.81 (1.97)	5.42 (1.11)	5.05 (1.23)	5.06 (1.45)	4.76 (1.44)
Standard + inoculation	4.85 (1.90)	3.86 (1.97)	5.49 (1.14)	5.13 (1.20)	5.09 (1.44)	4.82 (1.53)
Standard + narrative	4.94 (1.82)	3.89 (1.95)	5.42 (1.16)	5.11 (1.22)	5.13 (1.41)	4.85 (1.44)
Standard + inoculation + narrative	4.87 (1.86)	3.84 (1.87)	5.48 (1.13)	5.05 (1.22)	5.01 (1.46)	4.71 (1.50)
Health policy assignment						
20% tax on sugary drinks	4.49 (1.98)	3.53 (1.97)	5.52 (1.11)	5.08 (1.20)	5.31 (1.44)	4.82 (1.51)
Removal of sugary drink sponsorship from sport	5.20 (1.66)	3.80 (1.81)	5.61 (1.04)	4.93 (1.29)	4.91 (1.44)	4.34 (1.51)
Volume-based tax on alcohol	4.58 (1.93)	3.75 (2.03)	5.33 (1.16)	5.26 (1.12)	5.23 (1.35)	5.13 (1.37)
Removal of alcohol sponsorship from sport	5.18 (1.73)	4.07 (1.90)	5.36 (1.14)	4.97 (1.19)	4.85 (1.46)	4.71 (1.46)

Notes: M = mean; SD = standard deviation.

[a] Participants' level of support for their assigned policy which was recorded on a 7-point scale (1=*strongly oppose* to 7=*strongly support*).

[b] For participants assigned to a sugary drink policy, the anti-industry beliefs measured were that sugary drink companies: "deny that sugary drinks cause obesity"; "only care about making a lot of money"; "try to get young people to drink sugary drinks". For participants assigned to an alcohol policy, the anti-industry beliefs measured were that alcohol companies: "deny they market their products to young people"; "only care about making a lot of money"; "try to get young people to drink alcohol". Participants' level of agreement with their three anti-industry beliefs were recorded on 7-point scales (1=*strongly disagree* to 7=*strongly agree*), and subsequently averaged to create this outcome measure.

[c] For participants assigned to the "20% tax on sugary drinks" policy, the two non-targeted policies were removal of sugary drink sponsorship from sport and health warning labels on sugary drinks. For participants assigned to the "removal of sugary drink sponsorship from sport" policy, the two non-targeted policies were a 20% tax on sugary drinks and health warning labels on sugary drinks. For participants assigned to the "volume based tax on alcohol" policy, the two non-targeted policies were removal of alcohol sponsorship from sport and health warning labels on alcohol containers. For participants assigned to the "removal of alcohol sponsorship from sport" policy, the two non-targeted policies were a volume based tax on alcohol and health warning labels on alcohol containers. Participants' level of support for their two non-targeted policies were recorded on 7-point scales (1=*strongly oppose* to 7=*strongly support*), and subsequently averaged to create this outcome measure.
